# Scratch Where It Itches: Electronic Sharing of Health Information and Costs

**DOI:** 10.3390/healthcare11142023

**Published:** 2023-07-14

**Authors:** Na-Eun Cho, KiHoon Hong

**Affiliations:** College of Business, Hongik University, Seoul 04066, Republic of Korea; ncho@hongik.ac.kr

**Keywords:** challenges, health information technology, information sharing, hospital costs

## Abstract

The electronic sharing of health information holds the potential to enhance communication and coordination among hospitals and providers, ultimately leading to improved hospital performance. However, despite the benefits, hospitals often encounter significant challenges when it comes to sharing information with external parties. Our study aimed to identify the circumstances under which sharing information with external parties can result in changes in overall hospital costs, with a particular emphasis on various obstacles that hospitals may encounter, including lack of incentives or capabilities essential to facilitate effective information exchange. To achieve this goal, we obtain data from multiple sources, including the American Hospital Association (AHA) annual and IT surveys, the Center for Medicare and Medicaid Services (CMS) hospital compare dataset, and the Census Bureau’s small-area income and poverty estimates. Consistent with previous research, we observed a significant reduction in hospital costs when information was shared internally but not externally. However, our findings also revealed that the sharing of health information can lead to cost savings for hospitals when they encounter challenges such as the absence of incentives and capabilities regardless of whether the information is shared internally or externally. The implication of our study is simple but strong: perseverance and effort yield positive outcomes. Only when hospitals push through challenges related to sharing information can they achieve the anticipated advantages of information sharing. Based on our results, we suggest that policymakers should strategically target hospitals and providers that face challenges in sharing health information rather than focusing on those without obstacles. This targeted approach can significantly increase policy efficiency, and we emphasize the need for policymakers to address the specific areas where hospitals and providers encounter difficulties. By doing so, they can effectively “scratch where it itches” and address the core issues hindering the successful exchange of health information.

## 1. Introduction

Sharing information across different healthcare organizations and providers is known to offer numerous advantages such as enhanced coordination, communication, and knowledge about patients [1]. For instance, when information is shared, hospitals are likely to achieve economies of scale and operational complementarity, thus reducing costs of care [2,3,4]. Also, hospitals are known to improve the quality of care by reducing preventable readmissions and duplicate laboratory and radiology tests when they share patient health records and clinical information with multiple stakeholders [5,6,7]. The prospective benefits of health information sharing were the foundation for the enactment of the Health Information Technology for Economic and Clinical Health (HITECH) Act, which is the part of the American Recovery and Reinvestment Act of 2009.

Despite the overall success of the HITECH Act in promoting the widespread adoption and utilization of health information technology, it appears that not all hospitals and providers are fully recognizing the anticipated advantages of information sharing. The impact of sharing information differs depending on factors such as the organizational setting (e.g., teaching status, integrated healthcare systems, and ER crowdedness) and patient-related aspects (e.g., severity level) [8]. Additionally, a recent study revealed that the benefits of information sharing do not arise, particularly when organizations and providers share information with those outside the system compared to those within the system [9].

These findings indicate that hospitals and healthcare providers encounter diverse and nuanced challenges when it comes to facilitating efficient and successful information sharing across various organizations and providers. A comprehensive review of the existing literature provided valuable insights into numerous critical challenges that contribute to the intricate nature of information sharing in healthcare. These challenges encompass a range of factors, including limited technical capability to electronically send or receive information, obstacles in accurately identifying and matching patients across disparate systems, concerns related to billing and financial transactions, time constraints, difficulties in accessing accurate healthcare provider addresses, etc. [10,11,12]. These challenges underscore the intricacies involved in achieving successful information sharing within the healthcare system.

Our study built upon prior research by expanding on the finding that the benefits of sharing information are less apparent when sharing it with external entities compared to internal ones. We aimed to explore the factors that hinder the realization of these benefits by delving into the underlying circumstances. Specifically, our study aimed to identify the circumstances in which sharing information with those outside the system can lead to changes in overall hospital costs, with a particular emphasis on the difficulties and challenges that hospitals may encounter, such as the absence of incentives or capabilities.

To achieve this goal, we first collected data from multiple sources, including the American Hospital Association (AHA) annual and IT surveys, the Center for Medicare and Medicaid Services (CMS) hospital compare, and the Census Bureau’s small-area income and poverty estimates for the 2014–2016 period. We then estimated the association of information sharing and the costs of care at the hospital level. Similar to the previous literature [9], we found that the cost of care at the hospital level did not decrease when hospitals share information with those outside the system, while it did decrease with those inside the system. To our surprise, however, we found that the cost savings were salient when hospitals reported challenges such as the absence of incentives and capabilities regardless of whether they shared information with those inside and outside the system. 

The findings of our study have several implications worth noting. In terms of research, this study contributes to the existing body of literature [8,9] by deepening our understanding of the diverse contexts that either enhance or hinder the impact of sharing health information. While previous findings indicated that different systems may impede the realization of improved outcomes through information sharing, our study revealed that regardless of a hospital or provider’s system affiliation, the actual difficulties encountered during the process of sharing information serve as the primary determinants in generating the potential benefits. Therefore, it became evident that diligent efforts and perseverance can yield fruitful results.

For policy, our findings may help increase policy efficiency within limited resources. Policymakers should target hospitals facing challenges in ensuring information sharing rather than those without, as the ease of a task; that is, the lack of challenges, implies little room for improvement. The insignificant effects of sharing health information among hospitals that do not report challenges on cost savings suggest that they have already achieved the level at which the marginal benefit of sharing information with other stakeholders is only minimal. On the other hand, our results suggest that only hospitals that share information despite adversity can achieve the aforementioned benefits of sharing information. Using the findings from our study, policymakers can identify the groups in need and thus can direct resources toward those that are likely to have the grated impact; in other words, “Scratch where it itches”. 

The structure of the paper adheres to the following organization. In Section 2, we provide a detailed description of our data and model. Following that, in Section 3, we present the empirical results derived from our analysis and the discussion of these results. Finally, in Section 4, we offer the conclusions.

## 2. Materials and Methods

### 2.1. Data

We compiled data from multiple sources, including the American Hospital Association (AHA) annual and IT surveys, the Center for Medicare and Medicaid Services (CMS) hospital compare, and the Census Bureau’s small-area income and poverty estimates for the 2014–2016 period. The AHA conducts these surveys annually and collects information on health IT, including obstacles to exchanging health data, vendor type, degree of electronic transitions, etc., as well as a hospital’s general characteristics such as bed size, ownership type, payment, address, etc. The current study did not require review and approval from the institutional review board because the datasets utilized did not involve “human subjects”.

### 2.2. The Model

While alpha (α) represents the y-intercept or the constant term, (*β*) indicates the sensitivity of the dependent variable with regard to the independent variable.

$$\begin{array}{cc} {Hosptial\;Costs}_{it} & =\alpha +\beta_{1}\;{Information\;Sharing}_{it}\\ & +\;\beta_{2}{Bed\;Size}_{it}+\beta_{3}{For-profit\;Ownership}_{it}\\ & +\beta_{4}{Government\;Ownership}_{it}+\beta_{5}{Teaching\;Hospital}_{it}\\ & +\beta_{6}{Physician-Hospital\;Integration}_{it}\\ & +\beta_{7}{Capitation\;Revenue\;Ratio}_{it}\\ & +\beta_{8}{Urbanness}_{it}+\beta_{9}{HHI}_{it}\\ & +\beta_{10}{Median\;Household\;Income}_{it}+{Year}_{t}+\in i \end{array}$$

We collected our dependent variable (hospital costs) from the Medicare spending per beneficiary (MSPB) data obtained from the CMS hospital compare. This shows how much a hospital spends for an episode of care in relation to the national median. This is achieved by dividing each hospital’s expenditure by the median of the national episode-weighted expenditure. Therefore, the unit of hospital costs was not relevant. This calculation allowed us to determine whether each hospital spent more, less, or approximately the same amount for an episode of care compared to all hospitals nationally. Also, by considering patient age and health status as well as geographic payment differences, this measure allowed us to control for patient characteristics indirectly.

We obtained data on our independent variables *(information sharing within the system* and *information sharing outside the system)* by utilizing a question from the AHA IT surveys that asked, “Which of the following patient data does your hospital electronically exchange/share with one or more of the provider types listed below?” The responses to this question were used to generate the variables of *information sharing within the system* and *information sharing outside the system*. We aggregated the responses to the aforementioned question to determine the overall value of *information sharing within the system*: (1) with hospitals inside of the respondent’s system and (2) with ambulatory providers inside of the respondent’s system. For the variable of *information sharing outside the system*, we calculated the total value of *information sharing outside the system* by summing the responses to the aforementioned questions: (1) with hospitals outside of the respondent’s system and (2) with ambulatory providers outside of the respondent’s system. 

This implied that when the value of information sharing within the system was 0, the focal hospital did not electronically exchange information internally. Additionally, a value of 2 indicated that the focal hospital shared information electronically with both the hospital and the provider within the system. Similarly, a value of 1 suggested that they shared information with either the hospital or the provider internally. The same applied to the variable of information sharing outside the system. When the value was 0, it indicated that the focal hospital did not electronically exchange information with external parties. A value of 2 implied that the hospital shared information with both external hospitals and providers, and a value of 1 suggested that they shared information with either external hospitals or providers.

For our variables regarding difficulties that hospitals may face, we generated two binary variables: *capability-related challenges* and *incentive-related challenges*. The AHA IT survey also included the question, “Which of the following issues has your hospital experienced when trying to electronically (not eFax) send, receive or find (query) patient health information to/from other care settings or organizations? (Check all that apply)”. *Capability-related challenges* was a dummy variable that assumed a value of 1 when respondents checked one of the following: (1) We lack the technical capability to electronically send patient health information to outside providers or other sources; (2) We lack the technical capability to electronically receive patient health information from outside providers or other sources; (3) Providers we would like to electronically send patient health information to do not have an EHR or other electronic system with the capability to receive the information; and (4) Providers we would like to electronically send patient health information to have an EHR; however, it lacks the technical capability to receive the information. The variable took a value of 0 in all other cases. *Incentive-related challenges* was also a dummy variable that assumed a value of 1 when respondents checked one of the following: (1) Many recipients of our electronic care summaries (e.g., CCDA) report that the information is not useful; (2) Cumbersome workflow to send (not eFax) the information from our EHR system; (3) Difficult to match or identify the correct patient between systems; (4) Difficult to locate the address of the provider to send the information (e.g., lack of provider directory); (5) We have to pay additional costs to send/receive data with care settings/organizations outside our system; and (6) We do not typically share our patient data with care settings/organizations outside our system. The variable took a value of 0 in all other cases. 

Following the previous literature [4], we collected data on hospital or market characteristics from AHA annual and IT surveys and the U.S. Census Bureau’s small-area income and poverty estimates to adjust for the factors that may influence hospital costs. The measurement of bed size involved the utilization of eight predefined codes derived from the AHA annual survey. Each code corresponded to the following bed size categories: (1) 6–24 beds, (2) 25–49 beds, (3) 50–99 beds, (4) 100–199 beds, (5) 200–299 beds, (6) 300–399 beds, (7) 400–499 beds, and (8) 500 or more beds. For ownership type, we included two dummy variables: *for-profit ownership* and *government ownership*. This implied that when both dummies were zero, it was a voluntary nonprofit hospital. We also controlled for teaching status, physician–hospital integration, and capitation revenue ratio, following the prior literature [4]. *Teaching hospital* was a binary variable that assumed a value of 1 when the hospital was a teaching hospital, whereas *physician-hospital integration* was a dummy variable that assumed a value of 1 when a hospital employed an integrated salary model in which physicians were hired as employees and a value of 0 in all other cases [13,14]. *Capitation revenue ratio* represented the percentage of a hospital’s net revenue that was paid based on a fixed amount per patient for the provision of healthcare services. As control variables for market characteristics, we included *urbaneness*, the Herfindahl-Hirschman Index *(HHI)*, and *median household income*. The variable “*urbaneness*” was a binary indicator that assumed a value of 1 when the hospital was situated in an urban area and a value of 0 when it was located in a rural area. The *HHI* was calculated using the number of total facility admissions at the county level. While the variable representing *median household income* at the county level was sourced from the U.S. Census Bureau’s small-area income and poverty estimates, all the remaining control variables were collected from AHA surveys.

## 3. Results

### 3.1. Empirical Findings 

Table 1 presents descriptive statistics for a comprehensive set of 5947 hospital-year observations. Among these, the minimum hospital cost for an episode of care in relation to the national median was 0.61, whereas the maximum cost reached 1.70. Please note that hospital costs were measured by dividing each hospital’s expenditure by the median of the national episode-weighted expenditure. This indicated that while it should not necessarily have been exactly 1, the mean of the hospital cost variable should have been close to 1. Also, the average bed size of 4.592 indicated that it fell somewhere between the ranges of 100–199 beds and 200–299 beds. Our sample comprised a variety of hospital types. For example, 97% of the hospitals were general hospitals, while the remaining included specialty hospitals such as heart, obstetrics and gynecology, orthopedic, and rehabilitation hospitals, among others. Moreover, our sample consisted of diverse hospital ownership types, including nonprofit (including church-operated), for-profit (individual, partnership, and corporation), and government hospitals (state, county, city, etc.). In our sample, 67% of the hospitals were nonprofits, 17% were for-profits, and the remaining hospitals were government-owned. 

Among our sample, 6.17% of hospitals reported that they did not share information with hospitals and providers within the system, while 14.93% of them reported not sharing information outside the system. Also, 77% of hospitals indicated that either they themselves or the providers receiving electronic information from them lacked the necessary technical capabilities. Additionally, 63% of hospitals reported facing various challenges such as the need to pay fees, a lack of a culture of information sharing with others, or encountering complex procedures that demanded additional effort.

Before explaining the results of the following three tables, we would like to highlight that our standard errors were clustered at the hospital level to account for potential correlation within each hospital. By clustering the standard errors at this level, we aimed to avoid overestimating the precision of the estimates and to provide more robust inference in cases where clustering or dependence within our data existed.

Table 2 shows the main results of our ordinary least squares (OLS) regression analyses based on STATA software regarding the effect of information sharing within and outside the system. Consistent with previous studies, we found that the coefficient of information sharing was negative and statistically significant for information sharing within the system, indicating a reduction in costs. However, the coefficient of information sharing outside the system was negative but statistically insignificant, suggesting it did not have a substantial impact on costs. Please note that our results suggest that providers and hospitals can achieve greater cost savings by sharing information with more insiders because the variables for information sharing within and outside the system were not binary. However, sharing information with more external parties did not have a significant impact on cost savings.

Moreover, we observed a positive and statistically significant coefficient for bed size, which suggested the presence of diseconomies of scale rather than economies of scale. Additionally, the coefficient of for-profit hospital was positive and statistically significant, implying for-profit hospitals were more likely to offer services that are accompanied with the use of expensive equipment and procedures. The negative coefficient of teaching hospitals suggested that they can save costs compared to nonteaching hospitals. One possible explanation is that despite inconclusive findings as suggested by prior researchers [15,16,17], teaching hospitals may have had lower readmission rates and reduced follow-up costs, which ultimately resulted in lower overall costs compared to nonteaching hospitals [15]. The coefficients of physician–hospital integration and median household income were not statistically significant, implying that they did not significantly affect overall hospital costs. Moreover, the coefficient of capitation revenue ratio was negative and statistically significant. This indicated that providers under a capitation system, in which they receive a predetermined amount of payment per patient regardless of the actual services provided, had greater incentives to save costs compared to other revenue regimes. The positive coefficient of urbanness suggested that hospitals located in urban areas were more likely to offer costly services compared to those in rural areas. Lastly, the positive coefficient for HHI suggested that competition increased costs [18]. 

We conducted sub-sample analyses to observe whether the effect of information sharing with external parties varied with the existence of challenges, including lack of capabilities and incentives. First, we divided our sample into those that faced capability-related challenges (Table 3, column (1)) and those that did not (Table 3, column (2)). In addition, we divided our sample into those that faced incentive-related challenges (Table 3, column (3)) and those that did not (Table 3, column (4)).

Remarkably, we observed that the coefficients for information sharing outside the system were negative and statistically significant only in columns (1) and (3), while they were not significant in columns (2) and (4). These findings suggested that hospitals could effectively reduce costs through external information sharing only when they encountered substantial challenges related to capabilities and incentives. In other words, the prospective benefits of external information sharing could be realized by hospitals and providers solely when they overcame difficulties.

The results in Table 3 led us to further analyze whether the effect of information sharing within the system also varied with the existence of challenges, as shown in Table 4. As in Table 3, we divided our sample into those with capability-related challenges (Table 4, column (1)) and those without (Table 4, column (2)). In addition, we divided our sample into those with incentive-related challenges (Table 4, column (3)) and those without (Table 4, column (4)). As shown in Table 4, the coefficients of information sharing inside the system were negative and statistically significant only in columns (1) and (3) and not in columns (2) and (4). 

To our surprise, similar to what we found in Table 3, we found that the coefficients were negative and statistically significant only in columns (1) and (3) but statistically insignificant in columns (2) and (4). These results suggested that the presence of challenges played a key role in reducing hospital costs, not only when sharing information externally but also when sharing information internally.

### 3.2. Empirical Implications 

Our findings indicated that hospitals and providers could achieve cost reductions through electronic data sharing both within their system and with external parties, but only when they face and overcome challenges and difficulties. This raised a question regarding why previous studies have shown that sharing information with external parties outside the system did not lead to the expected benefits. One possible explanation is that the mixed effects observed in those studies created a lack of clarity. Alternatively, it is plausible that hospitals and providers within the same system were better prepared to address the challenges associated with data sharing by utilizing alternative approaches when necessary.

There are several implications that can be derived from our study. In terms of research, it enhances our understanding of the factors that contribute to the attainment of prospective benefits, such as cost reduction or quality improvement, resulting from sharing health information electronically [8,9]. While previous findings suggested that different systems hindered the attainment of better outcomes through information sharing [9], our study demonstrated that this is not always the case. Hospitals can still face difficulties when they share information internally, and conversely, hospitals may not encounter any challenges even when they share information externally. Our study highlighted that the actual difficulties encountered during the information-sharing process, irrespective of whether it occurs across the boundaries of hospitals or not, ultimately determine the potential benefits. We believe that this distinction constitutes a novel contribution to the existing literature. 

In terms of policy, our findings present opportunities to enhance policy efficiency. Specifically, policymakers should strategically target direct interventions toward hospitals that report challenges rather than implementing broad measures that encompass the entire population. Our findings strongly suggest that when challenges are scarce, the marginal effect of sharing information is minimal regardless of whether hospitals share information with those within or outside the system. More focus should be placed on addressing the actual obstacles and not the boundaries of hospitals. It is especially advisable to avoid solely relying on the previous literature and thus encouraging the sharing of information within the boundaries of hospitals. By focusing on those who face obstacles, policymakers can effectively target areas with the greatest potential for improvement. In other words, they should prioritize resolving the core issues and “scratch where it itches”.

Nevertheless, our study identified a few limitations that open avenues to future research. Firstly, despite our efforts to control for factors impacting hospital costs based on the available datasets, it is crucial to acknowledge the presence of other variables that may still influence costs but were not included in our analysis. Additionally, due to data limitations, we lacked specific information regarding the precise changes in test or procedure volumes that contributed to the observed cost savings. Future researchers can delve deeper into understanding whether the impact of information sharing on test or procedure volumes varies in relation to the challenges encountered, thereby providing an additional dimension to our study. Moreover, future studies could explore other types of challenges such as differences in vendor platforms or legal environments across states. Specifically, although not discussed in this paper, patient privacy concerns are one of the barriers to information sharing [19] in the era of big data and artificial intelligence (AI). Future researchers can also examine whether the existence of other challenges can also affect the impact of sharing information on costs. Lastly, as our primary dependent variable was hospital costs, future studies can explore other outcome variables such as length of stay, readmission rates, and more. This expanded exploration would further enhance our understanding of the complexities involved in information sharing and its potential effects on hospital costs.

## 4. Conclusions

The sharing of health information has been recognized as having the potential to improve communication among healthcare providers and hospitals, leading to cost reductions and improved patient outcomes. However, realizing these benefits can be challenging for some hospitals. Building upon previous research that highlighted the benefits of sharing information within the system but not with external parties, our study aimed to investigate the factors that hinder the attainment of these benefits with a specific focus on the challenges faced by hospitals, such as a lack of incentives or capabilities.

By utilizing a diverse range of data sources, including the American Hospital Association (AHA) annual and IT surveys, the Center for Medicare and Medicaid Services (CMS) hospital compare dataset, and the Census Bureau’s small-area income and poverty estimates for the period of 2014 to 2016, we found consistently across our results that hospitals and providers could effectively reduce costs through electronic data sharing whether within their own system or with external parties, but only when they confronted and overcame significant challenges.

The containment of overall hospital costs is of great interest to both researchers and policymakers. Our research highlighted the crucial significance of comprehending the diverse contexts in which the sharing of health information can either enhance or diminish its impact on cost savings. Furthermore, our findings emphasized a noteworthy opportunity to enhance policy efficiency by adopting a targeted approach. Instead of applying broad policies to the entire population, policymakers can focus their efforts on specific areas where hospitals and providers face challenges in electronically sharing health information. By addressing these specific pain points, policy interventions can lead to substantial cost savings. This approach would allow policymakers to “scratch where it itches,” addressing the precise areas where improvements are most needed and maximizing the impact of policy interventions. As a result, significant savings in policy costs can be achieved while effectively addressing the barriers to information sharing and reaping the benefits it can bring.

## Figures and Tables

**Table 1:** Descriptive Statistics.

Variable	Mean	SD	Min	Max
Hospital costs	0.984	0.076	0.610	1.700
Information sharing within the system	1.685	0.582	0	2
Information sharing outside the system	1.543	0.740	0	2
Bed size	4.592	1.850	1	8
For-profit ownership	0.175	0.380	0	1
Government ownership	0.154	0.361	0	1
Teaching hospital	0.095	0.294	0	1
Physician–hospital integration	0.475	0.499	0	1
Capitation revenue ratio	0.074	0.261	0	1
Urbanness	0.768	0.422	0	1
HHI	0.591	0.355	0.027	1
Median household income	55,067.800	14,500.170	24,001	134,609
Capability-related challenges	0.766	0.423	0	1
Incentive-related challenges	0.626	0.484	0	1

**Table 2:** The Impact of Information Sharing Within or Outside the System on Hospital Costs.

DV: Hospital Costs	(1)	(2)
Information sharing within the system	−0.007 ***	
	[0.003]	
Information sharing outside the system		−0.003
		[0.002]
Bed size	0.008 ***	0.008 ***
	[0.001]	[0.001]
For-profit hospital	0.039 ***	0.039 ***
	[0.004]	[0.004]
Government hospital	−0.005	−0.004
	[0.004]	[0.004]
Teaching	−0.014 ***	−0.013 ***
	[0.004]	[0.004]
Physician–hospital integration	−0.000	−0.001
	[0.003]	[0.003]
Capitation revenue ratio	−0.008 *	−0.008 **
	[0.004]	[0.004]
Urbanness	0.019 ***	0.018 ***
	[0.004]	[0.004]
HHI	−0.040 ***	−0.040 ***
	[0.005]	[0.005]
Median household income	−0.000	−0.000
	[0.000]	[0.000]
Constant	0.963 ***	0.956 ***
	[0.010]	[0.009]
Observations	5947	5947
R-squared	0.179	0.177

Standard errors (in brackets) are clustered at the hospital level; *** *p* < 0.01, ** *p* < 0.05, * *p* < 0.1; R-squared represents the proportion of the variance in the dependent variable.

**Table 3:** Impact of Information Sharing Outside the System in Relation to the Presence of Challenges.

DV: Hospital Costs	(1)	(2)	(3)	(4)
	Reporting of Capability-Related Challenges	Nonreporting of Capability-Related Challenges	Reporting of Incentive-Related Challenges	Nonreporting of Incentive-Related Challenges
Information sharing outside the system	−0.004 *	−0.002	−0.006 ***	−0.000
	[0.002]	[0.003]	[0.002]	[0.003]
Bed size	0.008 ***	0.008 ***	0.007 ***	0.009 ***
	[0.001]	[0.002]	[0.001]	[0.001]
For-profit hospital	0.036 ***	0.049 ***	0.039 ***	0.040 ***
	[0.004]	[0.008]	[0.004]	[0.005]
Government hospital	−0.005	−0.002	−0.001	−0.007
	[0.005]	[0.007]	[0.005]	[0.007]
Teaching	−0.015 ***	−0.008	−0.012 ***	−0.016 ***
	[0.004]	[0.007]	[0.004]	[0.006]
Physician–hospital Integration	−0.002	0.004	−0.000	−0.001
	[0.003]	[0.005]	[0.003]	[0.004]
Capitation revenue ratio	−0.010 **	−0.000	−0.013 ***	0.001
	[0.004]	[0.007]	[0.004]	[0.006]
Urbanness	0.018 ***	0.019 ***	0.024 ***	0.009
	[0.005]	[0.007]	[0.005]	[0.006]
HHI	−0.043 ***	−0.030 ***	−0.045 ***	−0.033 ***
	[0.005]	[0.009]	[0.005]	[0.008]
Median household income	0.000	−0.000	0.000	−0.000
	[0.000]	[0.000]	[0.000]	[0.000]
Constant	0.960 ***	0.947 ***	0.961 ***	0.953 ***
	[0.010]	[0.016]	[0.010]	[0.014]
Observations	4556	1391	3723	2224
R-squared	0.186	0.146	0.203	0.151

Standard errors (in brackets) are clustered at the hospital level; *** *p* < 0.01, ** *p* < 0.05, * *p* < 0.1.

**Table 4:** Impact of Information Sharing Within the System in Relation to the Presence of Challenges.

DV: Hospital Costs	(1)	(2)	(3)	(4)
	Reporting of Capability-Related Challenges	Nonreporting of Capability-Related Challenges	Reporting of Incentive-Related Challenges	Nonreporting of Incentive-Related Challenges
Information sharing within the system	−0.009 ***	−0.004	−0.009 ***	−0.006
	[0.003]	[0.004]	[0.003]	[0.004]
Bed size	0.009 ***	0.008 ***	0.008 ***	0.010 ***
	[0.001]	[0.002]	[0.001]	[0.001]
For-profit hospital	0.036 ***	0.048 ***	0.038 ***	0.039 ***
	[0.004]	[0.007]	[0.004]	[0.005]
Government hospital	−0.006	−0.002	−0.003	−0.008
	[0.005]	[0.007]	[0.005]	[0.007]
Teaching	−0.015 ***	−0.008	−0.012 ***	−0.016 ***
	[0.004]	[0.007]	[0.004]	[0.006]
Physician–hospital Integration	−0.002	0.005	−0.000	−0.001
	[0.003]	[0.005]	[0.003]	[0.004]
Capitation revenue ratio	−0.009 **	−0.000	−0.012 ***	0.001
	[0.004]	[0.007]	[0.004]	[0.006]
Urbanness	0.019 ***	0.019 ***	0.024 ***	0.010
	[0.005]	[0.007]	[0.005]	[0.006]
HHI	−0.043 ***	−0.031 ***	−0.046 ***	−0.032 ***
	[0.005]	[0.009]	[0.005]	[0.008]
Median household income	0.000	−0.000	0.000	−0.000
	[0.000]	[0.000]	[0.000]	[0.000]
Constant	0.968 ***	0.950 ***	0.968 ***	0.959 ***
	[0.011]	[0.017]	[0.012]	[0.014]
Observations	4556	1391	3723	2224
R-squared	0.189	0.147	0.204	0.153

Standard errors (in brackets) are clustered at the hospital level; *** *p* < 0.01, ** *p* < 0.05.

## Data Availability

The American Hospital Association (AHA) IT survey was purchased by N.C. The AHA annual survey data was provided by Jongwha Chang. Publicly accessible through the U.S. Census Bureau (https://www.census.gov, accessed on 15 February 2021), the small-area income and poverty estimates furnished information on median household income. Publicly available through Hospital Compare, we obtained data on “Medicare Spending Per Beneficiary—National,” which served as the source of information for our dependent variable (hospital costs). (https://data.cms.gov/provider-data, accessed on 1 April 2021).
